# Resolution enhancement of field asymmetric waveform ion mobility spectrometry (FAIMS) by ion focusing

**DOI:** 10.1186/1752-153X-7-120

**Published:** 2013-07-12

**Authors:** Fei Tang, Chulong Xu, Xiaohao Wang

**Affiliations:** 1 State Key Laboratory of Precision Measurement Technology and Instruments, Department of Precision Instrument, Tsinghua University, Beijing 100084, P.R. China

**Keywords:** Resolution, FAIMS, Ion focusing

## Abstract

**Background:**

Field Asymmetric Waveform Ion Mobility Spectrometry (FAIMS) is a material analysis technology which develops very fast in recent years. Resolution is an important factor used to estimate the performance of this technology. With greater resolution, it’s always easier to separate complex mixtures.

**Results:**

A method to increase the resolution of FAIMS is put forward which focuses ions before they enter the drift tube. By adding several pairs of focus electrodes loaded with DC or RF voltage in front of the FAIMS drift tube, the height of the ion beam flowing into the drift tube is decreased, which improves the resolution of the FAIMS spectrum. The effectiveness of this method is verified through SIMION simulation and experiments. Both the DC focusing mode and the AC focusing mode can improve the resolution of the FAIMS system, with the biggest increase of 37%.

**Conclusions:**

Compared with other methods of improving FAIMS resolution, this method needs neither additional special gases, nor additional auxiliary equipment. It is easy to miniaturize, and can work under atmospheric pressure.

## Background

### General background

With the social and economic development, the requirement for analysis technology has increased. The detection and analysis of Volatile Organic Compounds (VOCs) are extremely needed in the field of chemical weapons, explosives, drugs, pollutants [1]. People want the detection of VOCs to be fast, highly sensitive, and portable. Gas Chromatography and Mass Spectrometry are the most commonly applied technologies in the VOCs detection [2,3]. Traditional Gas Chromatography achieved VOCs detection at ppm level with the separation time of ten minutes level [4].Multidimensional Gas Chromatography improves the separation capacity of complex VOCs but the separation time is a bit longer [5]. The Mass Spectrometry technologies used in VOCs detection mainly include selected ion flow tube mass spectrometer (SIFTMS) [6], proton-transfer reaction-linear ion trap mass spectrometer (PTRMS) [7,8] and time of flight mass spectrometer (TOFMS) [9] which show a detection level of ppb and separation time with the range from seconds to several minutes. But Gas Chromatography or Mass Spectrometry can’t achieve on-site detection for its instrument volume or power consumption. Ion Mobility Spectrometry (IMS) is a very effective material analysis technology, which has a structure of several metal rings in a line arrangement, and shows a great potential to meet the requirement of detection for VOCs. However, IMS separates substances according to the differences in ion mobility between different materials. So when dealing with materials with similar ion mobility, its performance gets worse substantially. Field Asymmetric Waveform IMS (FAIMS) separates ions by using the change of ion mobility from low to high electric fields, and overcomes the shortcomings of IMS (in which substances with similar ion mobility cannot be separated). At the same time, the structure of FAIMS is simpler than IMS, so it is a promising material analysis technology for VOCs detection.

However, FAIMS technology is not yet mature, even after years of development, and one of its major problems is its lower resolution (than IMS). How to raise the resolution of FAIMS has become a hot area in FAIMS research. Currently, a number of ways to improve resolution have been developed. A cylindrical drift tube takes advantage of the inhomogeneity of the radial electric field, which has an effect of bringing specific ions towards a specific location together [10,11]; but the difficulties in cylindrical structure processing, concentricity in installation, and reduction of the drift tube (a structure of cylindrical or rectangular shape in which ions fly under the effect of the electric field) volume has limited its future development. The company Owlstone Nanotech in Great Britain has utilized MEMS technology to produce a 35 μm minimal-spacing drift tube, achieving high speed of analysis [12,13]. If Owlstone put the drift time to the normal level and high resolution would be obtained. However, owing to the tiny spacing, the power frequency needs to be over 20 MHz, which is a tough requirement for the power supply. Besides, because of the deep etching in the silicon wafer, it is difficult to control the roughness of the etching surface, which cannot be ignored given the 35 μm spacing, while the electrode flatness is a vital factor for the operation of the drift tube. Therefore, it is difficult to improve the test results of the separation with this tiny spacing design. Reducing the air pressure can also increase the resolution. With a lower pressure, the number of molecules per unit volume drops, which means an increase of *E/N* leading to an improvement of the resolution [14]. But an additional pump and other equipment will increase the system costs, which is inconsistent with the role of FAIMS as a “mass spectrum under atmospheric pressure”. A longer drift tube can also improve the resolution [15], but due to the long drift time (the time for ions to pass through the drift tube) through the drift tube, the ions will suffer severe losses and the signal intensity will be low. Besides, the long drift time make the diffusion can’t be ignored while short drift tube has no this problem [16]. Another way to improve resolution which is to inject He or H_2_ into the N_2_ carrier gas is put forward by Shvartsburg, and it has great effect in resolution improvement. [17,18] But, He and H_2_ gases are expensive, and this needs extra gas cylinders and pipings for the experiments, so it is not suitable for practical use in the future.

**Figure 1:**
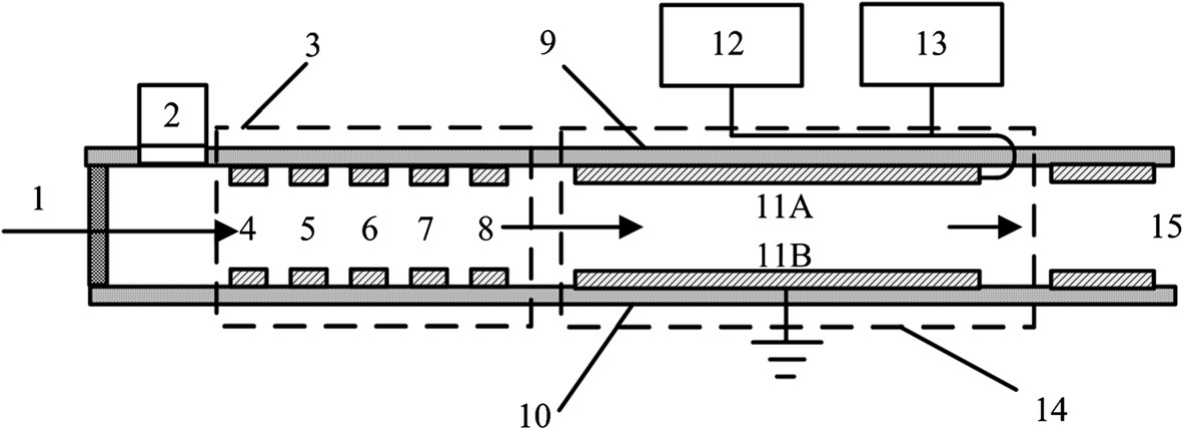
Diagram of the experimental focusing device: 1 - carrier gas; 2 - UV lamp; 3 -focus area; 4,5,6,7,8-focus electrode pairs; 9 - upper substrate plate; 10-lower substrate plate; 11A-upper electrode; 11B-lower electrode; 12 -DC scan compensation voltage; 13 - asymmetric waveform RF voltage; 14 - drift tube; 15 - detection unit.

In this paper, we come up with a novel method to improve resolution, by adding several pairs of focus electrodes in front of the planar FAIMS drift tube, as shown in Figure 1. This method combines FAIMS and ion focusing method such as ion lens and ion funnel together. The carrier gas 1 brings the sample into the ion mobility spectrometer, and the sample is ionized by UV lamp 2. When the ions are brought into the focus area 3 by the carrier gas, DC or AC voltages will be applied on the focus electrode pairs 4,5,6,7 and 8. The focus area forms an electric field to concentrate the ions to the center, so that the height of the ion beam entering the drift tube 14 is reduced, bringing about the improvement of the FAIMS resolution.

On upper electrode 11A, a DC scan compensation voltage 12 and an asymmetrical waveform RF voltage 13 is applied, and the lower electrode 11B is connected to the ground. The asymmetric wave form RF voltage 13 is a high-field asymmetric waveform with an average value of zero. The DC scan compensation voltage 12 is of a specific scanning frequency and range in a certain step. After being filtered in drift tube 14, the ions move to the right pulled by the carrier gas into the detection unit 15. The ions are deflected to the detection electrode by the deflection voltage, so the ion signals are converted into an electric current, which can be measured.

Currently, focusing of ions is mainly conducted in vacuum or a low pressure environment, and researches mainly focus on ion lens [19,20] and ion funnel [21-25]. In this paper, we extend the applications of these two focus methods to atmospheric conditions.

In an ion lens, various electrodes or rings are arranged in a certain configuration, with different electrostatic voltages applied, to form a transitional electric field in space which can control ion movement. Under vacuum conditions, the ion trajectory through an ion lens is similar to the trajectory of light through an optical lens, so “ion lens” is analogous to a lens technology. But, under atmospheric conditions, due to the interaction between gases and the ions, the electric field is no longer a conservative field, the law of ion movement does not agree with that under vacuum conditions. Through the SIMION simulation (in Results and Discussion), the focusing effect of ion lens under atmospheric conditions is verified. Then, the ion lens is used to the atmospheric condition in this paper.

The structure of an ion funnel is similar to that of an ion lens, but the voltage loaded on the electrode pairs is a high frequency sine wave voltage, and the difference between the voltage phases of adjacent electrode pairs is 180°. An ion funnel also can control the movement of ions, then create a focusing effect. Generally, an ion funnel works under low pressure conditions. In this paper, the use of ion funnel is extended to the atmospheric condition. The below SIMION simulation result also shows the focusing effect. For the pressure condition and focus structure is different, the simulation results of ion lens and ion funnel in this paper is different from that in the references.

### Theoretical background for ion focusing with FAIMS

The basic principle of FAIMS is ion separation based on the differences of ion mobility under high and low electric fields. The relationship between ion mobility and the electric field under the high field condition is

(1) $$K\left(E/N\right)=K_{0}\left[1+\alpha \left(E/N\right)\right].$$

The mobility of different ions under high electric field condition shows different non-linear changing trends, namely, *a*(*E/N*) differs for different ions, which allows ions with the same or similar mobility under low electric field condition be separated under high electric field condition. A planar FAIMS drift tube consists of two parallel electrodes. An asymmetrical square wave voltage is applied to one electrode. As ion mobility under high and low electric fields is different, ions will have a net displacement in the vertical direction in a single RF period, which differs according to the type of ion. With a DC compensation voltage added on the upper electrode at the same time, the net displacement of a particular ion in the vertical direction can be made up to 0, which will lead the particular ion to pass through the drift tube and be detected by the ion detector, while the other ions will be subject to annihilation when hitting the electrodes. By scanning the compensation voltage within a certain range, and recording the current values corresponding to the compensation voltage values, we can get a FAIMS spectrum.

In FAIMS, the resolution is defined as the ratio of the compensation voltage *U*_*M*_ corresponding to *I*_*max*_ in the FAIMS spectrum and the FWHM (Full width at half maximum), that is,

(2) $$R=U_{M}/\mathrm{FWHM}.$$

The compensation voltage *U*_*M*_ corresponding to the max current *I*_*max*_ in FAIMS spectrum is the DC compensation voltage value which makes ion’s net displacement zero in a RF cycle. FWHM is the difference between the compensation voltages at half of the peak value (when intensity is 0.5 *I*_*max*_ ). It can be seen that the FWHM has a large influence on the FAIMS resolution. The greater the FWHM, the lower the resolution. The reason why FWHM is non-zero is that when the compensation voltage deviates from *U*_*M*_, there are still ions that can fly through the drift tube and be detected, which have relationship with ions filling up the drift tube along the vertical direction.

**Figure 2:**
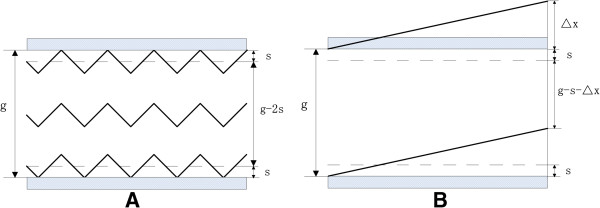
**Ions passing through the drift tube at the situation of no focusing when compensation voltage (A) is *U***_***M***_**(B) deviates from *U***_***M***_**.**

Assuming that the drift tube spacing is *g*, ions are uniformly distributed along the vertical direction of the drift tube, the vibration amplitude of ions under the asymmetric RF voltage is *s*, and the maximum current intensity for a specific ion can be measured when the compensation voltage is *U*_*M*_. At *U*_*M*_, the net displacement of the ions in an RF cycle in the vertical direction of the drift tube is exactly zero, as shown in Figure 2A. In most cases of short drift tube, the characteristic time of diffusion $t_{\mathrm{dif}}=g^{2}/\pi^{2}D(D$ is the diffusion coefficient) is always much larger than the drift time *t*_*res*_. That means the diffusion can be neglected due to the short drift time, which simplifies the problem [16].

The height of the ion beam at the entrance is g. However, due to the amplitude generated from ion vibration, the height of the ion beam that actually arrives at the exit of the drift tube is *g-2s*. Ions within a distance of s from the upper or lower electrodes are neutralized when hitting the drift tube. In other words, the area between the upper and lower counter electrodes is an ion annihilation region.

Now we consider the situation when the compensation voltage deviates from *U*_*M*_, as shown in Figure 2B. Assume that the compensation voltage is *U*. Denoting the net displacement of ions perpendicular to electrodes in the drift tube by *∆x*, the drift time by *t*_*res*_, and the mobility of ions in the low electric field by *K*_0_, then

(3) $$\mathrm{\Delta x}=\mathrm{abs}\left(U\;-\;U_{M}\right)/g\times K_{0}\times t_{\mathrm{res}}.$$

The height of the ion beam passing through the drift tube is g-s-*∆x*.

Assume that at some point the current intensity is half of its maximum value, so this point can be referred to as a “half peak position” point, and the corresponding net displacement is set as *∆x*_01_, then

(4) $$g\;-\;s\;-\;\Delta x_{01}=0.5\times \left(g\;-\;2s\right).$$

So $\Delta x_{01}=0.5\;g.$

Then $\mathrm{abs}\left(U\;-\;U_{M}\right)=\Delta x_{01}\times g/\left(K_{0}\times t_{\mathrm{res}}\right)=0.5\;g^{2}/\left(K_{0}\times t_{\text{res}}\right)$

So we can calculate that $\mathrm{FWHM}=2\mathrm{abs}\left(U\;-\;U_{M}\right)=2\Delta x_{01}\times g/\left(K_{0}\times t_{\mathrm{res}}\right)$, so ∆*x*_01_ can reflect the value of the FWHM, and also the resolution.

If ion focusing is applied, then the height of the ion beam entering the drift tube is smaller than the spacing of drift tube, which is denoted by *g*_*x*_.

(1) If *g* > *g*_*x*_ > *g* - 2*s.*

**Figure 3:**
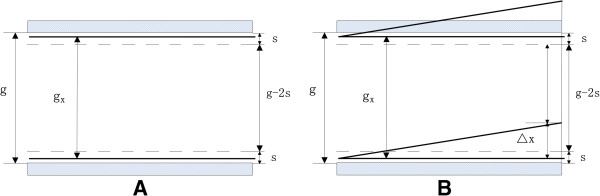
**Ions passing through the drift tube at the situation of**$g\;>\;g_{x}\;>\;g\;-\;2s$**when the compensation voltage (A) is *U***_***M***_**(B) deviates from *U***_***M***_***.***

First, the peak position will not change: when the compensation voltage is *U*_*M*_, the intensity will be the strongest, and the height of ion beam passing through is *g - 2s*, as shown in Figure 3A.

When the compensation voltage deviates from *U*_*M*_, the net displacement of ions perpendicular to electrodes in the drift tube is *∆x*, shown in Figure 3B.

Then the height of the ion beam passing through is *g*_*x*_ - *Δx* - [*s* - 0.5 × (*g* - *g*_*x*_)].

At the “half peak position” point, denote the net displacement by *∆x*_02_.

Then *g*_*x*_ - *Δx*_02_ - [*s* - 0.5 × (*g* - *g*_*x*_)] = 0.5 × (*g* - 2*s*).

So $\Delta x_{02}=0.5g_{x}<0.5g=\Delta x_{01}.$

At this time, *∆x*_02_ is less than the *∆x*_01_ without focusing, and the FWHM will be smaller. (2) If *g*_*x*_ < *g* - 2*s.*

**Figure 4:**
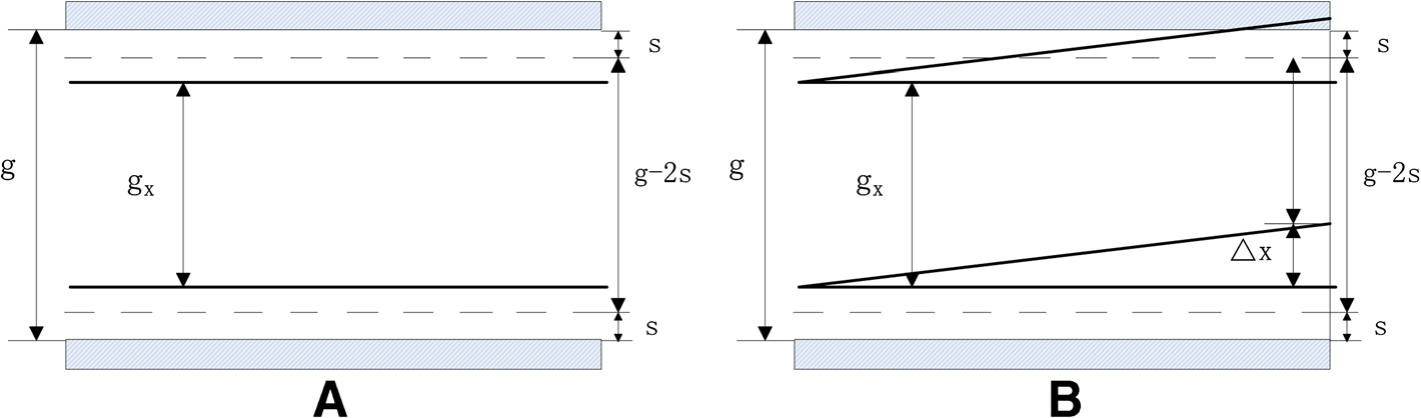
**Ions passing through the drift tube at the situation of**$g_{x}\;<\;g\;-\;2s$**when the compensation voltage (A) is *U***_***M***_**(B) deviates from *U***_***M***_***.***

At this occasion, the peak position will not change: when the compensation voltage is *U*_*M*_, the signal intensity will be the strongest, and the height of the ion beam passing through is *g*_*x*_, as shown in Figure 4A.

When the compensation voltage deviates from *U*_*M*_, shown in Figure 4B.

The width of the ion beam capable of passing through is: *g*_*x*_ - *Δx* + [0.5 × (*g* - *g*_*x*_) - *s*].

At the “half peak position” point, denote the net displacement by *∆x*_03_.

Then *g*_*x*_ - *Δx*_03_ + [0.5 × (*g* - *g*_*x*_) - *s*] = 0.5 × *g*_*x*_.

So $\Delta x_{03}=0.5g-s<0.5g=\Delta x_{01}$ and the FWHM will be smaller.

## Results and discussion

### SIMION simulation of ion focusing effect

After the above theory analysis, SIMION was used to conduct the simulation of the FAIMS resolution with ion focusing. For a drift tube with a spacing of 0.5 mm, the height of the entering ion beam is reduced from 0.48 mm to 0.40 mm. In every simulation, the ion intensity at the entrance is set as 100, and the ion intensity at the exit of the drift tube is recorded at different compensation voltage. The simulation results are shown in Table 1.

**Table 1:** Intensity corresponding to compensation voltage when the height of the entering ion beam is 0.48 mm/0.4 mm

											
CV	-7.4	-7.35	-7.3	-7.25	-7.2	-7.15	-7.1	-7.05	-7	-6.95	-6.9
0.48 mm	0	2	4	6	8	20	19	33	40	41	45
0.4 mm	0	0	4	10	15	19	21	39	32	44	41
CV	-6.85	-6.8	-6.75	-6.7	-6.65	-6.6	-6.55	-6.5	-6.45	-6.4	-6.35
0.48 mm	42	26	25	31	22	14	10	3	3	0	0
0.4 mm	42	40	32	23	16	13	6	1	4	0	0

When the height of the entering ion beam is 0.48 mm, the maximum intensity is 45, and the corresponding peak position is 6.9. After interpolation, it can be calculated that the half peak positions are -7.0875 and -6.55278, and the resolution is 6.9/(7.0875-6.55278) =12.9. On the other hand, when the height of the entering ion beam is 0.4 mm, the maximum intensity is 44, the corresponding peak position is 6.95, the half peak positions are -7.09722 and -6.70556, and the resolution is 6.95/(7.09722-6.70556)=17.7.

It can be seen that after the height of the entering ion beam is constrained, the resolution has been improved. The improvement in the resolution is mainly due to the decrease in the FWHM. Therefore, focusing the ion beam entering the drift tube and reducing its height can help to improve the resolution of the system and enhance the performance of the instrument.

### SIMION simulation of ion focusing methods

Through the above theoretical analysis and simulations, it can be seen that focusing the ion beam entering the drift tube can help to improve the resolution. In order to focus the ion beam, we extend certain focusing methods such as ion lens and ion funnel to atmospheric condition.

**Figure 5:**
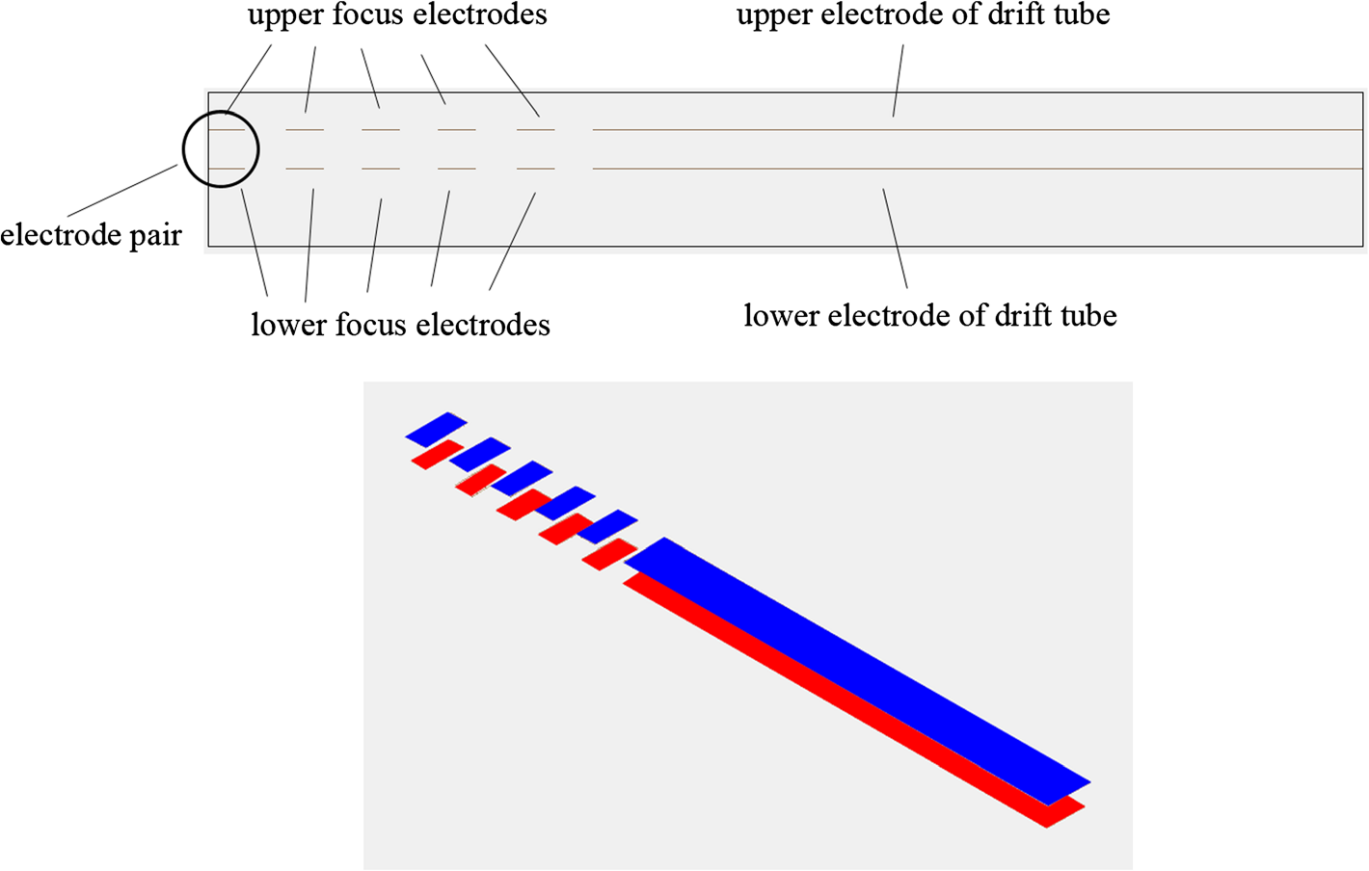
Structure of focusing electrode pairs: the upper electrodes are blue and the lower electrodes are red.

The focus structure built in SIMION is designed as shown in Figure 5. In front of the drift tube, five pairs of parallel electrode pairs are added to form a focus area, and voltages will be applied to focus the ions.

#### ***Simulation results of ion lens***

Under atmospheric conditions, we use the collision_sds database in the SIMION program to simulate ion trajectories.

**Figure 6:**
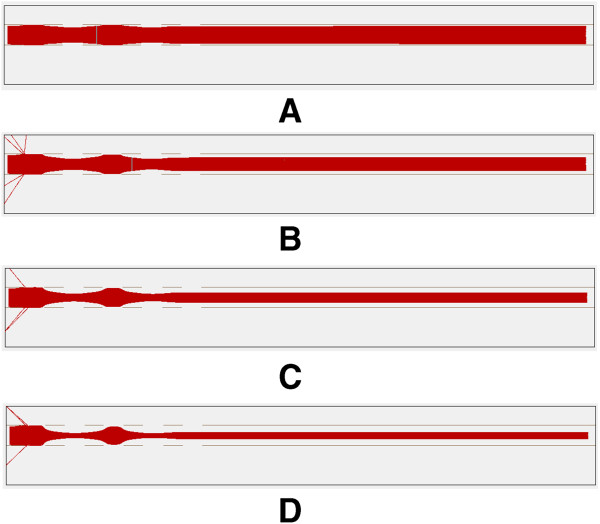
**Ion trajectories under different static voltage conditions.** Voltages on electrode pairs from left to right: **(A)** 0–1.25V-0-1.25V-0 **(B)** 0–2.5V-0-2.5V-0 **(C)** 0–3.75V-0- 3.75V-0 **(D)** 0-5V-0-5V-0.

Figure 6 shows the trajectories of ions moving through focusing electrode pairs under different static voltages. Table 2 shows the relevant data of the ion movement under different voltages. The position of the upper ion beam and the lower ion beam represent the highest and lowest locations of the ions after they pass through the focusing area, and the zero point of the vertical coordinate is set at the plane of the lower electrode in the focusing area. It can be seen that with higher voltage, the ion beam gets more focused and narrowed.

**Table 2:** Data of Ion movement under different voltages

**Voltage loaded**	**Position of upper ion beam/mm**	**Position of lower ion beam/mm**
0-1.25V-0-1.25V-0	0.4738	0.0125
0-2.5V-0-2.5V-0	0.4323	0.0863
0-3.75V-0-3.75V-0	0.3863	0.1329
0-5V-0-5V-0	0.3473	0.1720

#### ***Simulation results of ion funnel***

Here, we also use the collision_sds database of the SIMION program to simulate ion trajectories in an ion funnel under atmospheric conditions. The simulation was conducted under two conditions: one is under the same frequency with different peak-to-peak voltages, and the other is under the same peak-to-peak voltage with different frequencies.

a) Simulation under the same frequency with different peak-to-peak voltages.

**Figure 7:**
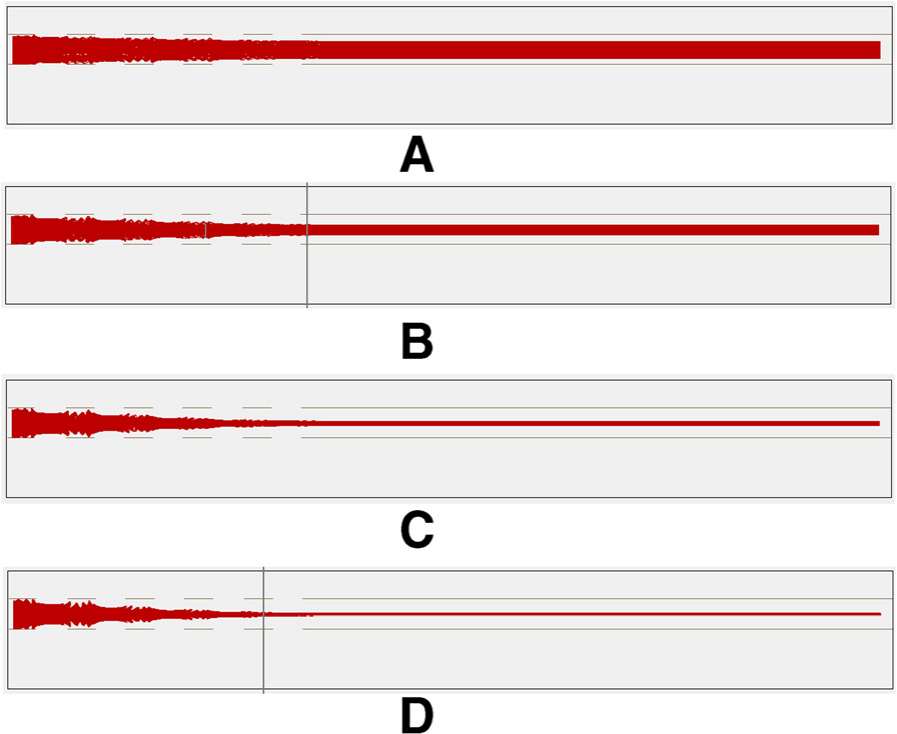
Trajectories of ions under the same frequency with different peak-to-peak voltages: (A) 25 kHz 20 Vpp (B) 25 kHz 40 Vpp (C) 25 kHz 60 Vpp (D) 25 kHz 80 Vpp.

Figure 7 shows the trajectories of ions under the same frequency with different voltages. And Table 3 shows the relevant data of ion movement. Under the same frequency conditions, the higher the AC focus voltage, the smaller the width of the ion beam.

b) Simulation under the same peak-to-peak voltage with different frequencies.

**Figure 8:**
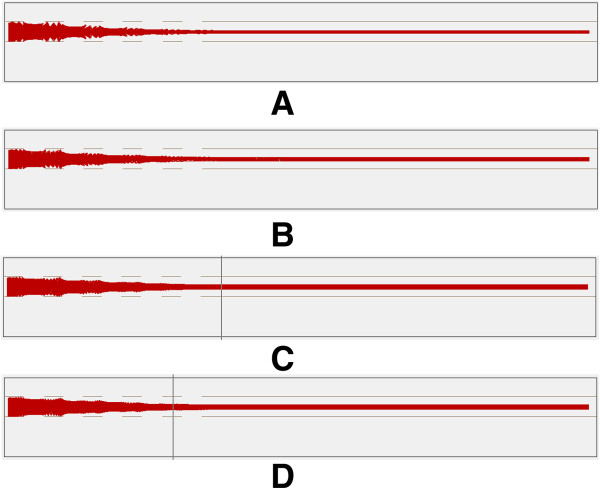
Trajectories of ions under the same peak-to-peak voltage with different frequencies: (A) 20 kHz 60 Vpp (B) 30 kHz 60 Vpp (C) 40 kHz 60 Vpp (D) 50 kHz 60 Vpp.

**Figure 9:**
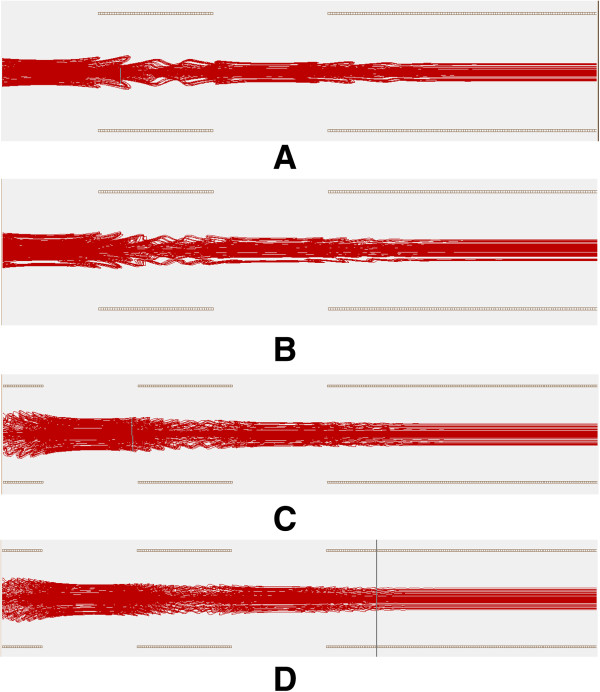
Details of ions leaving the focus area: (A) 20 kHz 60 Vpp (B) 30 kHz 60 Vpp (C) 40 kHz 60 Vpp (D) 50 kHz 60 Vpp.

Figure 8 shows the trajectories of ion movement under the same peak-to-peak voltage with different frequencies. Table 4 shows the relevant data of ion movement. From the simulation results at first sight, it seems that frequency does not have a large impact on the focusing effect. However, when we have a look at the details of ions leaving the focus area as shown in Figure 9, we can see that with the decrease of frequency, the direction of ions moving into the drift tube is no longer horizontal. The ions have a trend to diverge, which means the poorer focusing effect. And this will lead to the increase of FWHM and is verified in the later experiment.

**Table 3:** Data of Ion movement under the same frequency and different peak-to-peak voltages

**Voltage loaded**	**Position of upper ion beam/mm**	**Position of lower ion beam/mm**
25 kHz 20 Vpp	0.4147	0.1079
25 kHz 40 Vpp	0.3495	0.1623
25 kHz 60 Vpp	0.2993	0.2126
25 kHz 80 Vpp	0.2870	0.2433

**Table 4:** Data of Ion movement under the same peak-to-peak voltage and different frequencies

**Voltage loaded**	**Position of upper ion beam/mm**	**Position of lower ion beam/mm**
20 kHz 60 Vpp	0.3281	0.1832
30 kHz 60 Vpp	0.3206	0.2028
40 kHz 60 Vpp	0.3241	0.1931
50 kHz 60 Vpp	0.3220	0.1832

Compared with an ion lens, an ion funnel can achieve a better focusing effect, and the ions are more concentrated. Overall, both the ion lens and ion funnel have an obvious focusing effect on ions going through the focus area. For simplicity, we will call the ion lens method the “DC focus” mode, and the ion funnel method the “AC focus” mode.

### Experiment of FAIMS chip with ion focusing

After the simulation of ion focusing effect and ion focusing methods, the experiment of FAIMS chips with ion focusing is conducted. In the experiments, nitrogen containing 10 ppm of acetone is adopted as the sample gas, and the flow rate is 0.96 L/min., the power supply frequency of the asymmetric square wave is 1 MHz, and the duty cycle is 30%. The detection mode of positive ions is adopted. The diffusion time is about 2 ms while the drift time is about 0.2 ms in this experiment.

#### ***DC focus mode***

**Figure 10:**
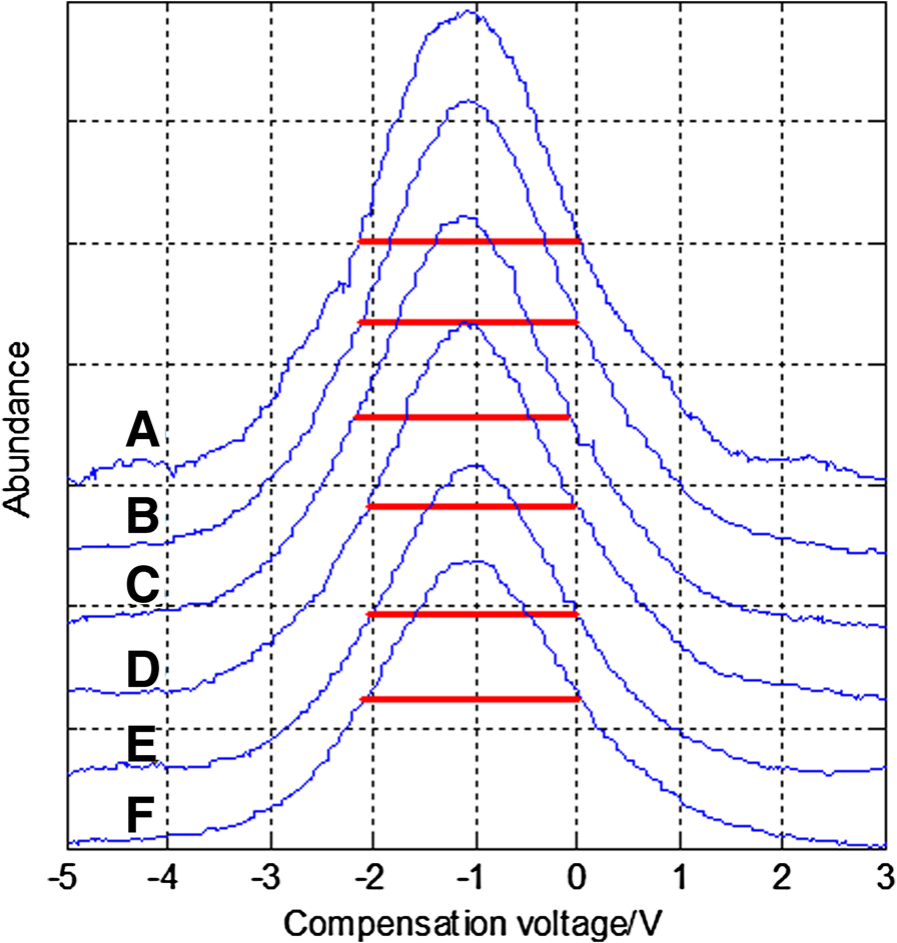
Spectrum of acetone under different DC focus voltages when peak-to-peak asymmetric waveform RF voltage is 850 V: (A) no focus (B) 0–2.5V-0-2.5V-0 (C) 0-6V-0-6V-0 (D) 0-9V-0-9V-0 (E) 0-15V-0-15V-0 (F) 0-21V-0-21V-0.

**Figure 11:**
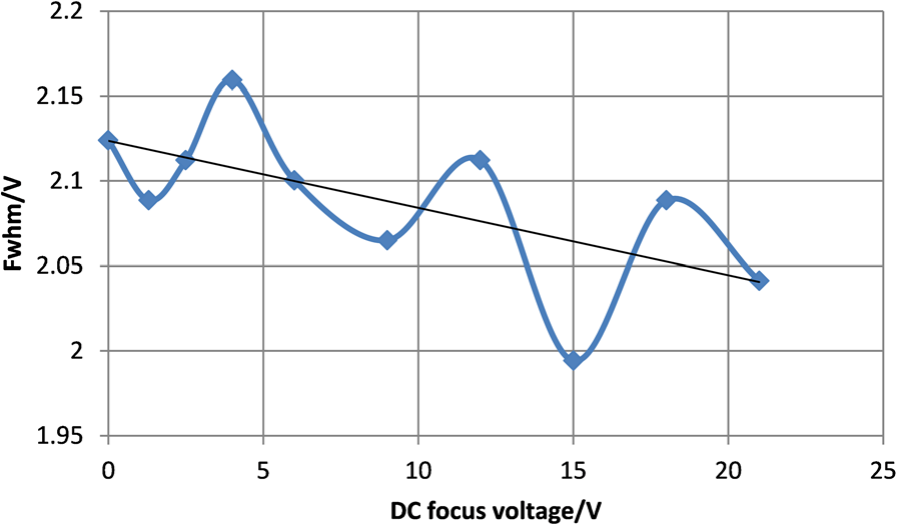
FWHM of acetone under different DC focus voltages when the peak-to-peak asymmetrical waveform RF voltage is 850 V.

Figure 10 shows the spectrum of acetone under different DC focus voltages when peak-to-peak asymmetric waveform RF voltage is 850 V while the red dash line shows the FWHM. Each spectrum under different focus voltages is torn apart to be seen more clearly. And Figure 11 shows the change of FWHM with the focus voltage. When the peak-to-peak asymmetric waveform RF voltage is 850 V, DC focus can improve the resolution. The best focusing effect is achieved when the DC focus voltage is about 15 V: the FWHM is reduced by 2.124-1.9942=0.1298 V, and the resolution is enhanced by 2.124/1.9942-1=6.5%. In general, the FWHM decreases with higher voltage. The focusing effect is not very strong and there are points that don’t obey the rule. A possible reason is that the positive and negative ions of acetone are both generated by the UV lamp. In DC focus mode, focusing the positive ions means a divergence of the negative ions, so the interaction between the positive and negative ions can lead to a lessened focusing effect.

#### ***AC focus mode***

a) Same focus frequency with different focus voltages.

**Figure 12:**
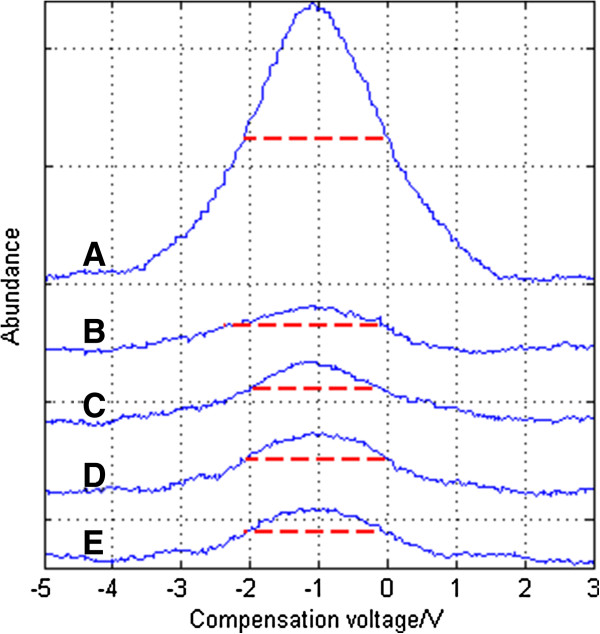
Spectrum of acetone under different RF focus voltages when peak-to-peak asymmetric waveform RF voltage is 850 V: (A) no focus (B) 25 kHz 12 Vpp (C) 25 kHz 30 Vpp (D) 25 kHz 49 Vpp (E) 25 kHz 60 Vpp.

**Figure 13:**
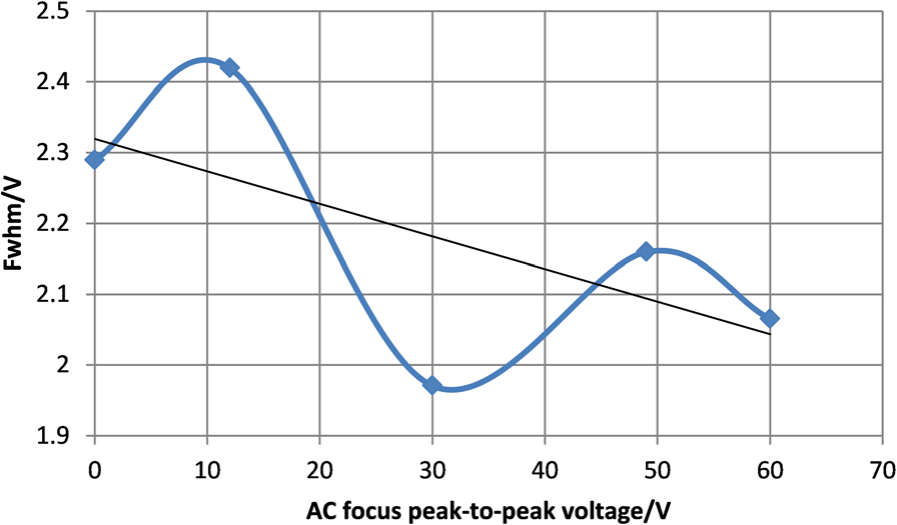
FWHM of acetone under different RF focus voltages when the peak-to-peak asymmetrical waveform RF voltage is 850 V.

In AC focus mode, the spectrum is shown in Figure 12 and it can be seen that the focusing effect is larger than that under DC focus mode, but the signal intensity is lower. It’s mainly because that ions vibrate more in AC focus mode than DC focus mode and ion losses is bigger. When the asymmetric waveform RF voltage is 850 V, an RF focus voltage at a peak-to-peak value of 30 V achieves the best focusing effect: the FWHM is reduced by 2.2892-1.9706 = 0.3186 V, and the resolution is enhanced by 2.2892/1.9706-1 = 16.17%, as shown in Figure 13. The “ion funnel” has focusing effects on both the positive and negative ions, so resolution enhancement is bigger than that under DC focus mode. But the compounding and collision between positive and negative ions can lead to more complex conditions than where there is only one single kind of ion, so there are still experimental points with an ineffective focusing effect.

b) Same focus voltage with different focus frequencies.

**Figure 14:**
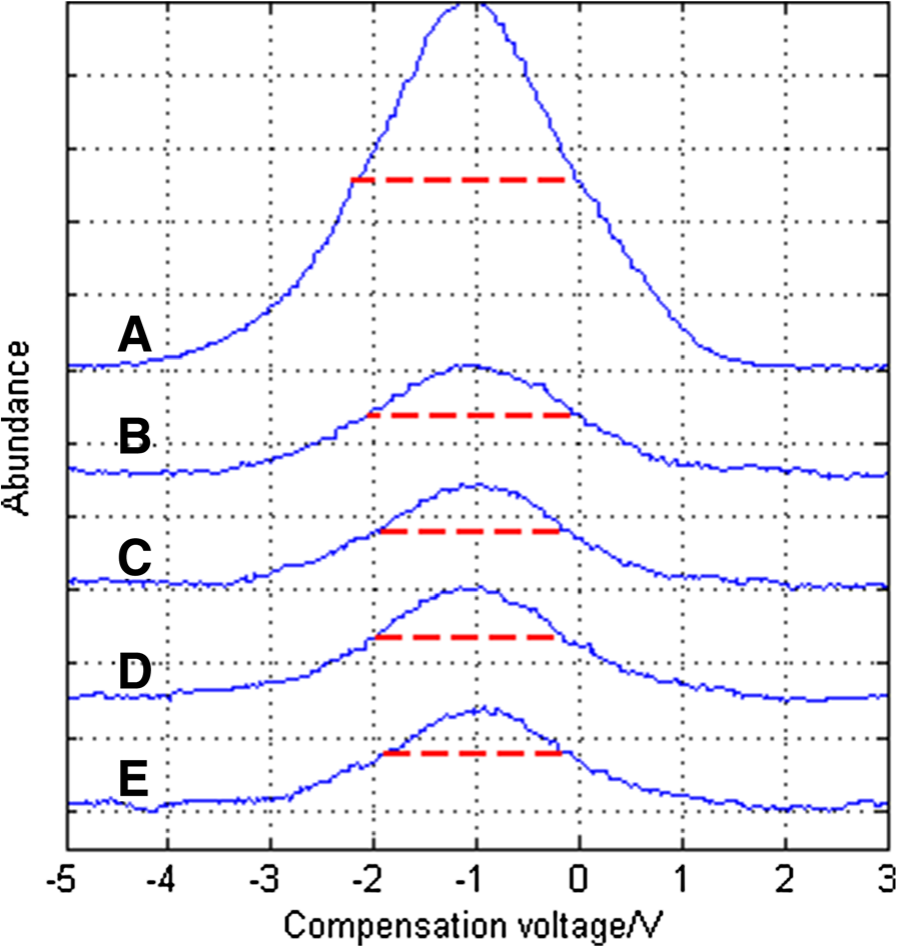
Spectrum of acetone under different RF focus voltage frequencies when peak-to-peak asymmetric waveform RF voltage is 800 V: (A) no focus (B) 20 kHz 60 Vpp (C) 30 kHz 60 Vpp (D) 40 kHz 60 Vpp (E) 50 kHz 60 Vpp.

**Figure 15:**
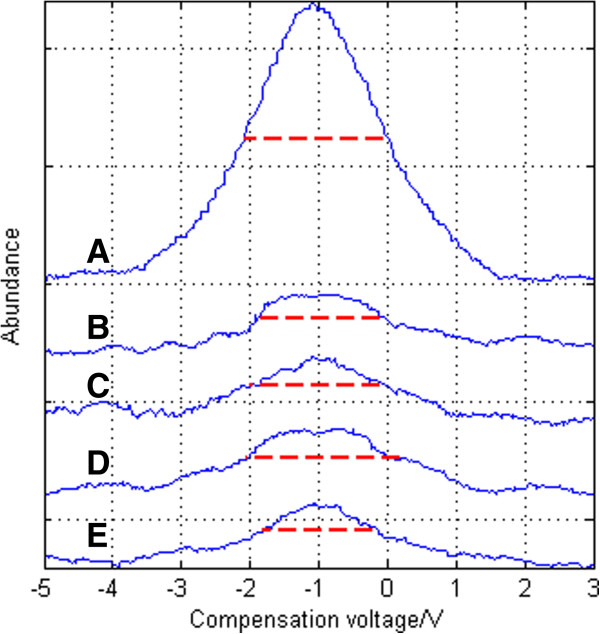
Spectrum of acetone under different RF focus voltage frequencies when peak-to-peak asymmetric waveform RF voltage is 850 V: (A) no focus (B) 20 kHz 60 Vpp (C) 30 kHz 60 Vpp (D) 40 kHz 60 Vpp (E) 50 kHz 60 Vpp.

**Figure 16:**
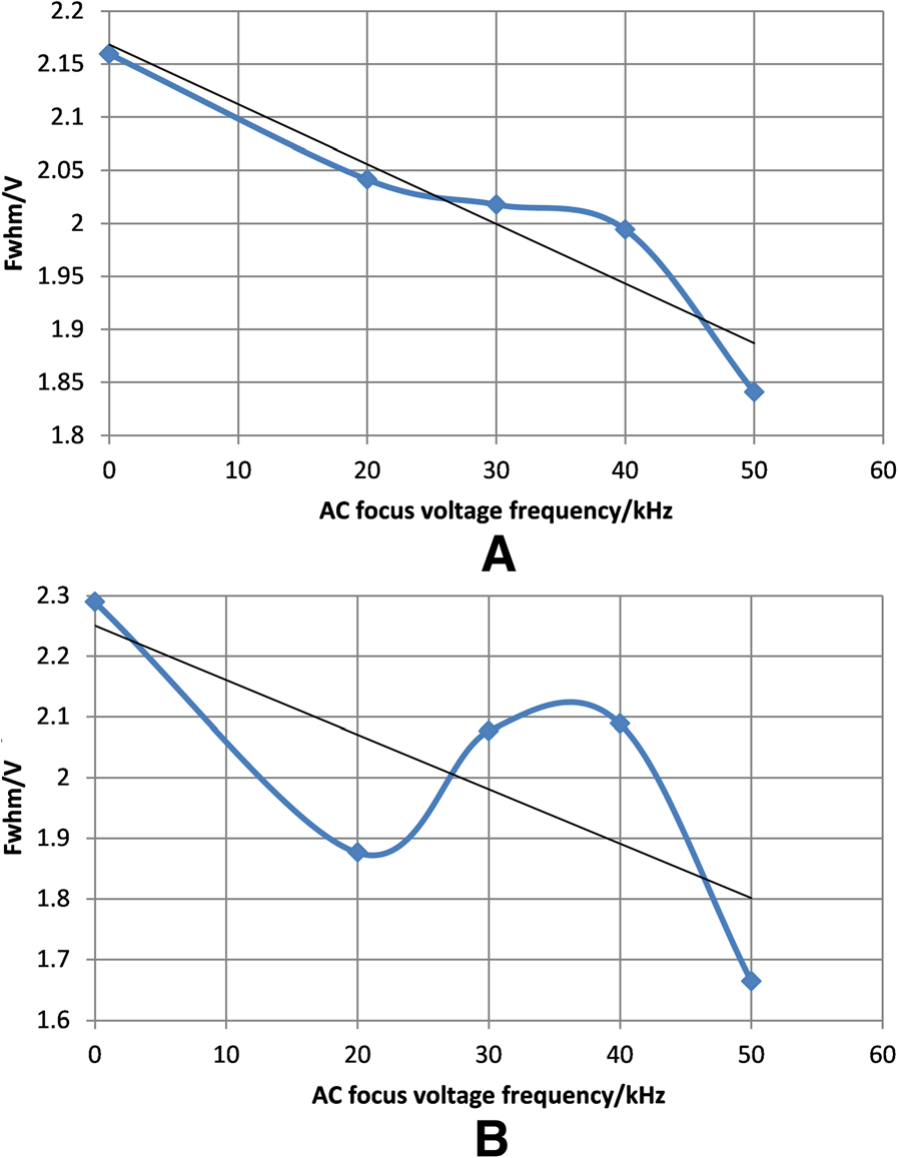
FWHM of acetone under different RF focus voltage frequencies when the peak-to-peak asymmetrical waveform RF voltage is (A) 800 V and (B) 850 V.

The spectrum of the experiment of adjusting the frequency is shown in Figure 14 and Figure 15. Under asymmetrical waveform RF voltages of both 800 V and 850 V, it is seen that the best effects can be achieved under 50 kHz. Under asymmetric waveform RF voltage of 800 V, the FWHM is reduced by 2.1594-1.8408 = 0.3186 V, and resolution is enhanced by 2.1594/1.8408-1 = 17.3%, as shown in Figure 16A; under asymmetric waveform RF voltage of 850 V, the FWHM is reduced by 2.2892-1.6638 = 0.6254 V, and resolution is enhanced by 2.2892/1.6638-1 = 37.59%, as shown in Figure 16B. The experiment shows that FWHM has the trend of decreasing with the increase of frequency. In the part of “Simulation under the same peak-to-peak voltage with different frequencies” of this paper, the simulation result shows with smaller frequency, focusing effect becomes poorer and ions have a diverging trend which may lead to the increase of FWHM. This agrees with the experiment result.

### Experimental

#### ***Parameters of ion focusing effect simulation***

In the simulation of ion focusing effect, SIMION was used to simulate the change of FAIMS resolution when the height of the entering ion beam is reduced from 0.48 mm to 0.40 mm. The drift tube consists of two planar electrodes with a spacing of 0.5 mm and a length of 15 mm. The other parameters are laid out: the maximum RF voltage is 690 V, duty cycle is 30%, frequency is 1 MHz, gas flow speed is 6.667 m/s, and *a*(*E/N*) of the ion is 0.046.

#### ***Parameters of ion focusing methods simulation***

In the simulation of ion focusing methods, the focus structure is shown in Figure 5. In front of the drift tube, five pairs of parallel electrode pairs are added to form a focus area. The spacing of the drift tube is 0.5 mm and the length is 10 mm. The spacing between upper and lower focusing electrode is also 0.5 mm. The length of each focusing electrode is 0.5 mm and the interval between pairs of electrodes is 0.5 mm. The gas flow speed is 3.6 m/s and the ions are acetone ions.

#### ***Instrumentation of FAIMS chip experiment***

To verify the focusing effect, the experiment conducts the FAIMS chip with ion focusing conducted in the experiment was designed and manufactured, as shown in Figure 1.

**Figure 17:**
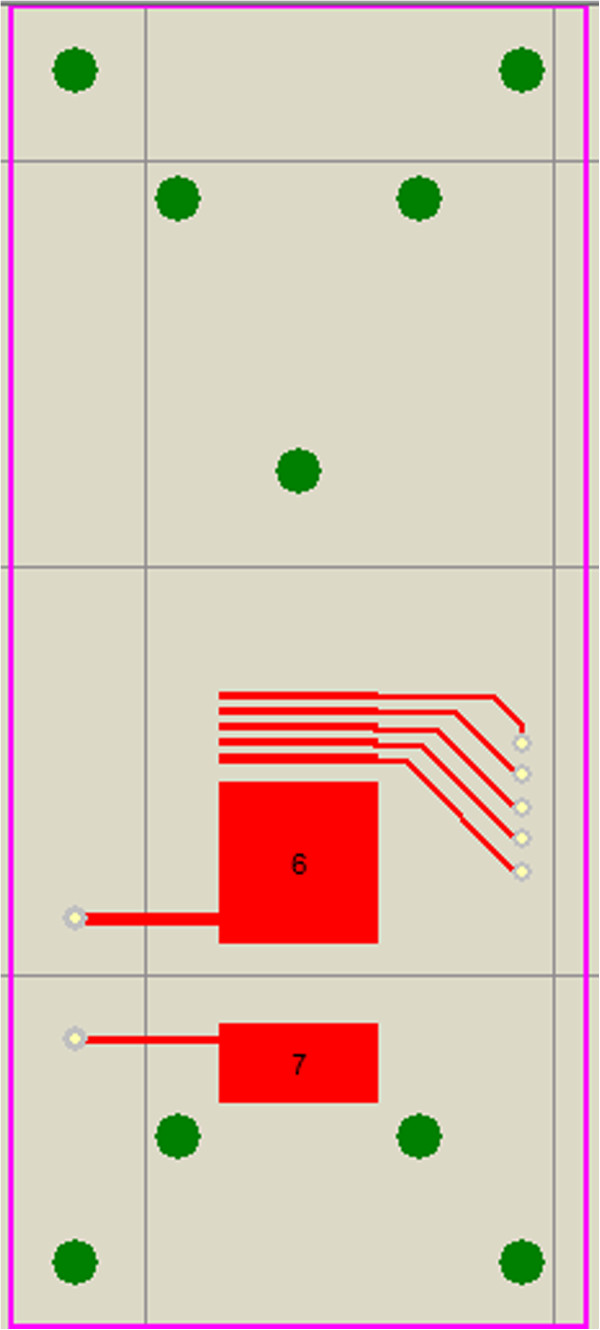
PCB layout of the upper substrate plate in FAIMS chip with ion focusing.

**Figure 18:**
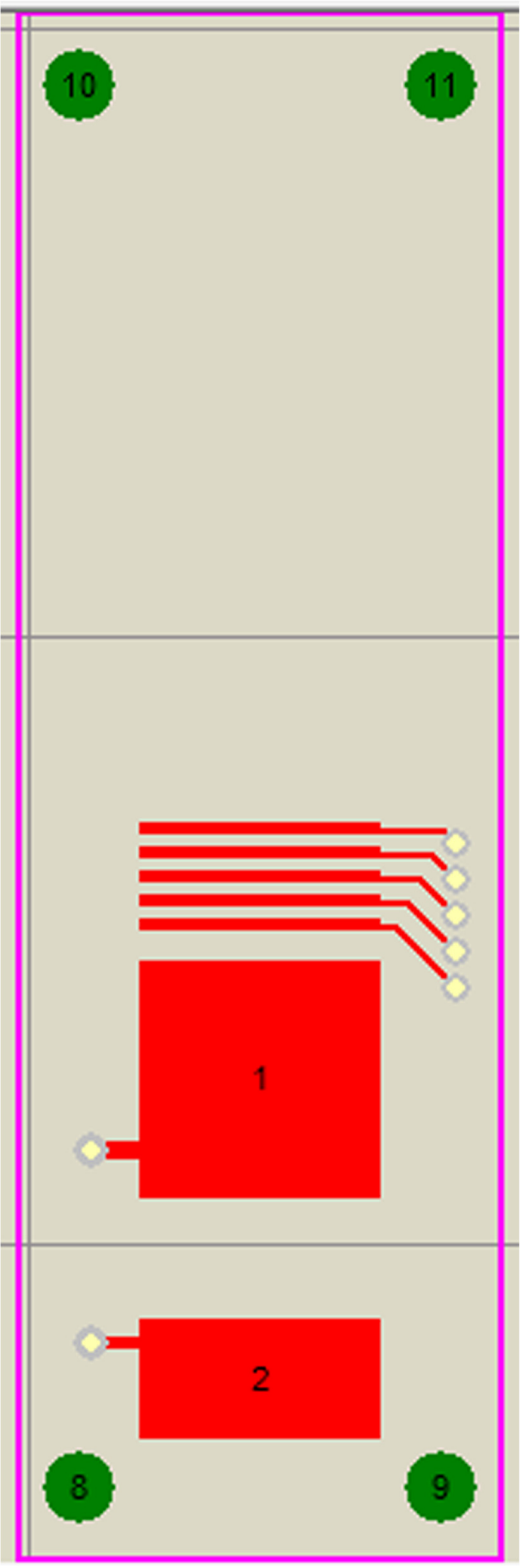
PCB layout of the lower substrate plate in FAIMS chip with ion focusing.

PCB board is adopted as the substrate plate of the device; rectangular pads are placed on the circuit board as electrodes. The PCB layout of upper substrate plate is shown in Figure 17 and the PCB layout of lower substrate plate is shown in Figure 18. The width of all electrodes is 10 mm. The length of focus electrodes is 0.5 mm and the interval between them is also 0.5 mm. The length of the drift tube is 10 mm. The length of detection unit electrodes is 5 mm. All geometries are confirmed by slide caliper. The PTFE plates with the height of 0.5 mm are placed in the middle between PCB boards to define the spacing of the drift tube, and the device is sealed with silicone rubber. A UV lamp is used as the ion source, and is positioned above the through-hole of the chip in the middle of upper substrate plate.

Other instrument used in the experiment is laid out as follow: we used a bottle of nitrogen containing 10 ppm acetone (volume of 8 L, pressure of 10 MPa, Beijing Hua Yuan Gas Chemical Co., Ltd) to bring the sample into the FAIMS chip. D08-1F-type flow indicator (Beijing Seven Star Electronics Co., Ltd.) is used to control the gas flow. The DC scan compensation voltage source and asymmetric waveform RF voltage source are homemade. The DC scan compensation voltage source can provide a voltage changing from -15 V to +15 V with a step of 0.1 V. The asymmetric RF waveform source can provide a rectangular asymmetric waveform voltage with peak-to-peak value of 2000 V, frequency of 1 MHz, duty cycle of 30%.

## Conclusions

After adding a focusing structure in the front of drift tube loaded with a DC or AC voltage, the height of the ion beam entering into the drift tube is narrowed, leading to a decrease of FWHM and improvement of the resolution. Both the DC focus mode and AC focus mode can improve the resolution. The resolution is increased by 37% at the most under the AC mode. Further study to improve the resolution and stability can be carried out from the perspective of removing anisotropic ions. Overall, this innovation of focus is simple in structure and easy to miniaturize. The DC and AC voltages are easy to obtain, without increasing the complexity of the system. In addition, this method has no special requirements on the sample material or carrier gas, and can be used under atmospheric pressure, so it has the potential for wide range of applications. Besides, this ion focusing method will broad the way of FAIMS resolution enhancement and might cooperate with other method of increasing resolution. FAIMS works on the base of ion behavior under electric field. The design of the electric field plays an important role in FAIMS performance and will receive more and more attention in the lateral FAIMS research.

## Competing interests

The authors have no competing interests to declare.

## Authors’ contributions

TF carried out the theoretical analysis and the computational experiments. XC carried out the structure design and the focusing experiments. WX conceived of the study, and participated in its design and helped to draft the manuscript. All authors read and approved the final manuscript.
